# HIV Infection and the Epidemiology of Invasive Pneumococcal Disease (IPD) in South African Adults and Older Children Prior to the Introduction of a Pneumococcal Conjugate Vaccine (PCV)

**DOI:** 10.1371/journal.pone.0149104

**Published:** 2016-02-10

**Authors:** Susan Meiring, Cheryl Cohen, Vanessa Quan, Linda de Gouveia, Charles Feldman, Alan Karstaedt, Keith P. Klugman, Shabir A. Madhi, Helene Rabie, Charlotte Sriruttan, Anne von Gottberg, GERMS-SA

**Affiliations:** 1 Division of Public Health Surveillance and Response, National Institute for Communicable Diseases of the National Health Laboratory Service, Johannesburg, South Africa; 2 Centre for Respiratory Diseases and Meningitis, National Institute for Communicable Diseases, a division of the National Health Laboratory Service, Johannesburg, South Africa; 3 School of Public Health, Faculty of Health Sciences, University of the Witwatersrand, Johannesburg, South Africa; 4 Division of Pulmonology, Department of Internal Medicine, Charlotte Maxeke Johannesburg Academic Hospital and Faculty of Health Sciences, University of the Witwatersrand, Johannesburg, South Africa; 5 Department of Internal Medicine, Chris Hani Baragwanath Academic Hospital, Johannesburg, South Africa; 6 Department of Global Health, Hubert School of Public Health, Emory University, Atlanta, GA, United States of America; 7 Medical Research Council: Respiratory and Meningeal Pathogens Research Unit, School of Pathology, University of the Witwatersrand, Johannesburg, South Africa; 8 Department of Science and Technology/National Research Foundation: Vaccine Preventable Diseases, University of the Witwatersrand, Johannesburg, South Africa; 9 Department of Pediatric Medicine, Tygerberg Hospital, Cape Town, South Africa; 10 Centre for Opportunistic, Tropical and Hospital-associated Infections, National Institute for Communicable Diseases, a division of the National Health Laboratory Service, Johannesburg, South Africa; Faculdade de Medicina de Lisboa, PORTUGAL

## Abstract

**Introduction:**

*Streptococcus pneumoniae* is the commonest cause of bacteremic pneumonia among HIV-infected persons. As more countries with high HIV prevalence are implementing infant pneumococcal conjugate vaccine (PCV) programs, we aimed to describe the baseline clinical characteristics of adult invasive pneumococcal disease (IPD) in the pre-PCV era in South Africa in order to interpret potential indirect effects following vaccine use.

**Methods:**

National, active, laboratory-based surveillance for IPD was conducted in South Africa from 1 January 2003 through 31 December 2008. At 25 enhanced surveillance (ES) hospital sites, clinical data, including HIV serostatus, were collected from IPD patients ≥ 5 years of age. We compared the clinical characteristics of individuals with IPD in those HIV-infected and -uninfected using multivariable analysis. PCV was introduced into the routine South African Expanded Program on Immunization (EPI) in 2009.

**Results:**

In South Africa, from 2003–2008, 17 604 cases of IPD occurred amongst persons ≥ 5 years of age, with an average incidence of 7 cases per 100 000 person-years. Against a national HIV-prevalence of 18%, 89% (4190/4734) of IPD patients from ES sites were HIV-infected. IPD incidence in HIV-infected individuals is 43 times higher than in HIV-uninfected persons (52 per 100 000 vs. 1.2 per 100 000), with a peak in the HIV-infected elderly population of 237 per 100 000 persons. Most HIV-infected individuals presented with bacteremia (74%, 3 091/4 190). HIV-uninfected individuals were older; and had more chronic conditions (excluding HIV) than HIV-infected persons (39% (210/544) vs. 19% (790/4190), p<0.001). During the pre-PCV immunization era in South Africa, 71% of serotypes amongst HIV-infected persons were covered by PCV13 vs. 73% amongst HIV-uninfected persons, p = 0.4, OR 0.9 (CI 0.7–1.1).

**Conclusion:**

Seventy to eighty-five percent of adult IPD in the pre-PCV era were vaccine serotypes and 93% of cases had recognized risk factors (including HIV-infection) for pneumococcal vaccination. These data describe the epidemiology of IPD amongst HIV-infected and -uninfected adults during the pre-PCV era and provide a robust baseline to calculate the indirect effect of PCV in future studies.

## Introduction

*Streptococcus pneumoniae* is the most common cause of bacteremia and bacterial pneumonia among HIV-infected persons, and has been shown to occur between 30 and 100 times more frequently in HIV-infected than HIV-uninfected individuals.[1–4] Recurrent disease is seen more frequently with HIV infection, with approximately 25% of patients having a recurrence within 12 months.[1, 5, 6]

This study was conducted in South Africa, which reports a HIV prevalence of 18% amongst adults in 2008.[7] In population groups with a high HIV prevalence the epidemiological pattern of invasive pneumococcal disease (IPD) seems to change, with a peak seen in the young adult population as well as the typical but more marked peaks seen in the infant and elderly population groups.[4, 6, 8] Despite the higher rate of IPD in HIV-infected individuals the majority of studies have not shown higher case-fatality ratios amongst people co-infected with HIV.[1, 3, 6]

Some differences in IPD epidemiology have been described in HIV-infected versus HIV-uninfected individuals. Firstly, HIV-infected adults tend to be infected more commonly with pneumococcal serotypes that are typically seen in young children, and in relation to this, HIV-infected people tend to have a higher proportion of penicillin non-susceptible isolates.[3, 9–12] In contrast, serotype 1 disease tends to be proportionally less common amongst HIV-infected than HIV-uninfected persons.[3, 4, 13] Besides HIV as a stand-alone risk factor, HIV-infected individuals with IPD tend to have less underlying disease than HIV-uninfected individuals.[14]

Use of the polysaccharide pneumococcal vaccine (PPV23) in HIV-infected African adults is controversial.[5] However, studies using the 7-valent pneumococcal conjugate vaccine (PCV7) are promising, showing a 74% vaccine efficacy against vaccine serotypes in HIV-infected adults with a previous episode of IPD, albeit with subsequent waning of immunity.[15] Some countries, including South Africa, have already shown an indirect effect in the adult population following infant PCV immunization.[16–18] In South Africa and the United States of America, this decrease was seen in both HIV-infected (-25 to -59% for vaccine serotypes attributed to childhood PCV immunization) and HIV-uninfected adults (-52 to -86% for vaccine serotypes).[18, 19]

Reporting baseline clinical characteristics of IPD amongst adults in a setting with high HIV prevalence is important to elicit the indirect and long-term effects, including serotype replacement, of PCV introduction on IPD in adult populations. In this analysis we aimed to describe the baseline epidemiology of IPD in the pre-PCV era amongst HIV-infected and -uninfected persons ≥ 5 years of age using data derived from national surveillance.

## Methods

GERMS-SA conducts national, active laboratory-based surveillance across South Africa in a network consisting of approximately 130 public and private microbiology laboratories. Each laboratory is responsible for sending *S*. *pneumoniae* isolates, along with demographic details of the patient to a reference laboratory (the Centre for Respiratory Diseases and Meningitis (CRDM) at the National Institute for Communicable Diseases (NICD)) where confirmatory tests, serotyping and antimicrobial susceptibility testing are performed. Data from individuals diagnosed with IPD from 1 January 2003 through to 31 December 2008 were included in this analysis. From 2003 to 2005 our surveillance network strengthened and remained relatively stable thereafter. For this reason trends in incidence by serotype were only examined from 2005 onwards. Comprehensive audits of cases identified but not reported were conducted for all the public laboratories: unreported cases ranged from 16% to 21% over the 6 years and their demographic data were included in our database.[20] PCV7 was introduced into the South African EPI in 2009, and replaced by PCV13 in 2011. There was minimal use of either PCV7 or PPV23 during the study period.

IPD was defined as identification of *S*. *pneumoniae* from any usually sterile site (cerebrospinal fluid (CSF), blood, pleural fluid, joint fluid, ascitic fluid, vitreous fluid, etc.). Multiple isolates of *S*. *pneumoniae* identified from the same person were counted as one case provided they occurred within 21 days of the first isolate. *S*. *pneumoniae* were identified by either: culture of the organism, consistent Gram stain plus detection of *S*. *pneumoniae* antigen by latex agglutination, or detection of *S*. *pneumoniae* by direct PCR (polymerase chain reaction) of the specimen. Antimicrobial disc diffusion susceptibility testing of potentially resistant strains was confirmed by broth microdilution. Antimicrobial susceptibility was reported as susceptible or non-susceptible (intermediate and resistant isolates) based on the Clinical and Laboratory Standards Institute (CLSI) interpretive criteria.[21] Serotyping of viable organisms were determined using the Quellung reaction (Statens Serum Institut, Copenhagen, Denmark). Serotypes were grouped according to those covered by PCV13 (1, 3, 4, 5, 6A, 6B, 7F, 9V, 14, 18C, 19A, 19F and 23F) and PPV23 vaccines (1, 2, 3, 4, 5, 6B, 7F, 8, 9N, 9V, 10A, 11A, 12F, 14, 15B, 17F, 18C, 19A, 19F, 20, 22F, 23F, 33F and serotype 6A cross-protection was assumed). When calculating incidence of individual serotypes we assumed the age-specific proportion of serotypes with non-viable or missing pneumococcal isolates to be that of those with available isolates and imputed serotype data for 5 041 cases.

Enhanced surveillance (ES) was conducted at 25 sentinel-hospital sites situated in all 9 provinces of South Africa. Where available, consenting persons with IPD (or their legal guardians) were interviewed using a standardized questionnaire. Clinical details were retrieved from medical record review performed on cases of IPD at these hospitals. As clinical details were only available from patients presenting to ES sites, a comparison of cases from enhanced versus non-enhanced surveillance sites was done to show representativeness of data from the ES sites.

The following demographic and clinical details were collected from each person under surveillance: age/ date of birth, sex and specimen type (CSF, blood, other for any other specimen from a sterile site). Additional details were collected at ES sites: clinical syndrome (meningitis, bacteremic pneumonia, bacteremia without focus, other), Pitt bacteremia score for severity of illness (score 0 for mild, 1–3 for moderate, 4–12 for severe illness),[22] HIV status, in-hospital outcome and any underlying condition predisposing to IPD. Predisposing conditions were further classified into ACIP (Advisory Committee on Immunization Practices) conditions (asplenia/ sickle cell disease; chronic lung, renal, liver or cardiac condition; diabetes; solid organ transplant; immunotherapy; malignancy; primary immunodeficiency; CSF leak following a head injury; alcohol dependency; tobacco use) and other risk factors (cerebrovascular accident; burns).[23] Demographic, laboratory and clinical details for each patient were linked using Epi Info, version 6.04d, and later updated to Access.

Incidence of IPD was calculated using South African population denominators from Statistics-South Africa.[24] HIV-specific incidence rates for South Africa were calculated by assuming that the proportion of patients who tested positive for HIV at sentinel sites was the same in those who were not tested. HIV population denominators for 2008 were obtained from the Thembisa model 1.7 version 3.[25]

Statistical analysis was done using Stata version 11 (StataCorp Inc., College Station, Texas, USA) and p-values of ≤0.05 were considered significant throughout. Trends in incidence rates by age-category and serotype were calculated using Poisson regression. Univariate analysis of characteristics associated with IPD among HIV-infected and HIV-uninfected persons was performed using Fisher’s exact test or the Mantel–Haenszel χ^*2*^-test for categorical variables. Multivariable logistic regression models were evaluated comparing IPD cases at enhanced and non-enhanced surveillance sites, and comparing IPD cases amongst those HIV-infected and–uninfected. We started with all variables that were significant at p-value less than 0.05 on univariate analysis, and dropped non-significant factors with stepwise backward selection. All two-way interactions were evaluated.

Ethical clearance and permission to conduct laboratory-based and enhanced surveillance in South Africa for this study was obtained from the Health Research Ethics Committee (Human), University of Witwatersrand (Clearance number M02-10-42); the University of Stellenbosch Health Research Ethics Committee (Reference number N04/01/0021), the National Institute for Communicable Diseases Research Committee (Clearance number M060449); and the South African Department of Health (Reference H2/12/8). The following consent procedures were approved by the above mentioned ethics committees: Written consent was obtained from all patients (or parents/legal guardians of minors) at ES sites, prior to being interviewed, for participation in the ES program. Patients from ES sites who had already been discharged from hospital were interviewed telephonically and gave verbal consent, documented by surveillance officers on the written consent forms. Parents/ legal guardians of minors gave written consent (or verbal if the child had already been discharged from hospital) on behalf of their children <18 years of age. Patients from ES sites who were unable to provide written or verbal consent (due to severity of illness, death or lost to follow up) were still included in the study and their clinical data were collected only through medical record review. Participants not consenting to answer the enhanced surveillance questionnaire were still included in the laboratory-based surveillance program. All patient identifiers were removed prior to data analysis.

## Results

### Surveillance Population

**Fig 1:**
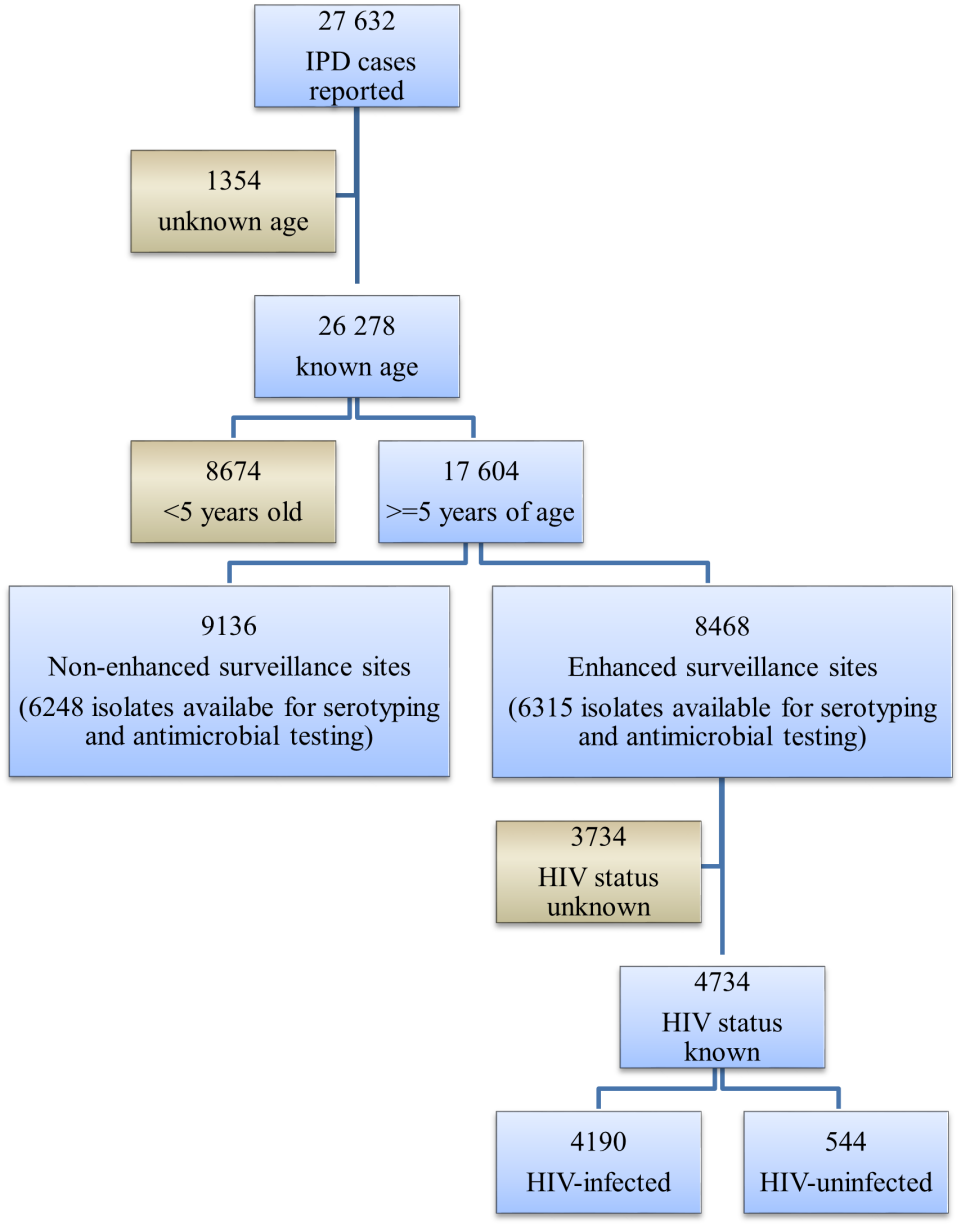
Flow diagram of cases of invasive pneumococcal disease, South Africa, 2003–2008 (n = 27 632).

From 2003 through 2008, 27 632 individuals with IPD were reported to GERMS-SA surveillance. Ninety-five percent (26 278) had known age, and 17 604 (67%) of these were aged 5 years and older. Antimicrobial susceptibility testing and serotyping were performed on 71% (12 563/17 604) of the isolates. Forty-eight percent of individuals (8 468/17 604) were from ES sites. Medical records were found for 82% (6 960/8 468) of cases at ES sites and data on HIV-serostatus were available for 68% (4 734) of these individuals. (Fig 1)

### Incidence of invasive pneumococcal disease

**Fig 2:**
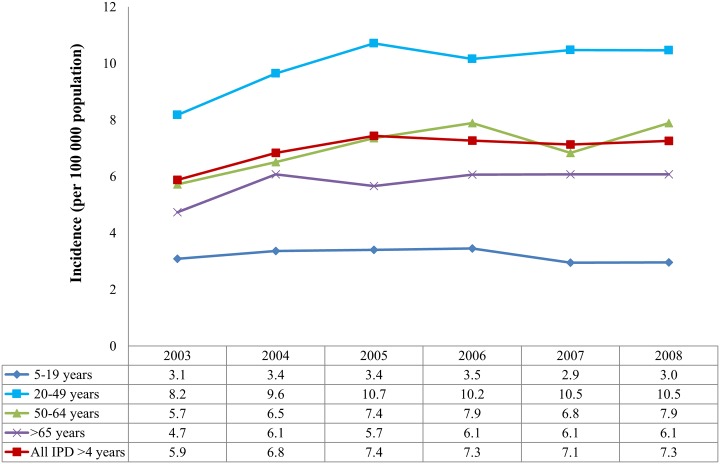
Incidence of invasive pneumococcal disease by age category and year, South Africa, 2003–2008 (n = 17 604). Footnote: Between 2003 and 2004 there was a significant increase in disease (p<0.001), most notably in the 20–49 year age group (p<0.001), however from 2005 to 2008 disease incidence did not change significantly overall, however a small decrease was noted in the 5–19 year age group (p = 0.004).

Incidence of IPD occurring in persons ≥ 5 years of age increased from 2003–2005, then remained relatively stable (7.4 to 7.3 per 100 000 population, p = 0.27; 95% CI 0.98–1.00) from 2005–2008. A small decrease in incidence was seen in the 5–19 year age group from 2005–2008 (3.4 to 3 per 100 000 population, p = 0.004, 95% CI 0.91–0.98). (Fig 2)

**Fig 3:**
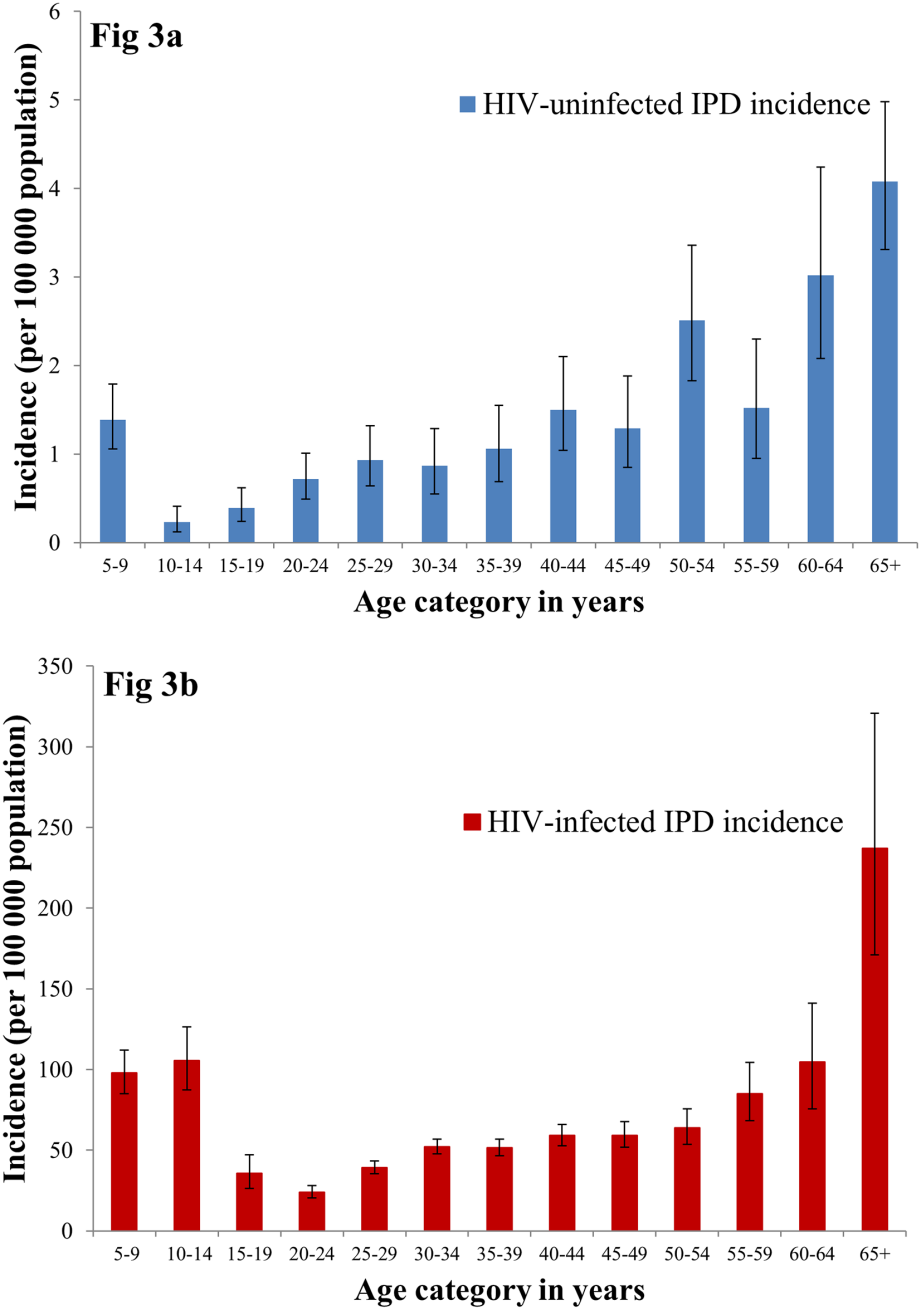
Incidence of invasive pneumococcal disease (IPD) amongst HIV-uninfected and HIV-infected persons by age category, South Africa, 2008.

In 2008, the relative risk of IPD amongst HIV-infected individuals was 43 times greater than HIV-uninfected individuals (52 per 100 000 vs. 1.2 per 100 000, respectively). (Table 1) For HIV-uninfected individuals, incidence increased with increasing age, with a peak in those ≥ 65 years (4.1 per 100 000). (Fig 3a) However, in HIV-infected individuals incidence followed a U-shaped curve with peaks in the later childhood and elderly age categories (81.4 in 5–19 years; 46.3 in 20–49 years; 74.7 in 50–64 years; 237.2 in ≥65 years). (Fig 3b; Table 1)

**Table 1:** Annual incidence and relative risk of invasive pneumococcal disease amongst HIV- infected and -uninfected persons by age category, South Africa, 2008.

Age category	Invasive pneumococcal disease incidence						Relative Risk	(95% confidence interval)
	Overall (95% confidence interval)		HIV-infected (95% confidence interval)		HIV-uninfected (95% confidence interval)			
5–19yrs	3.2	(3–3.5)	81.4	(73.4–90)	0.6	(0.5–0.8)	127	(101–160)
20–49yrs	10.1	(9.7–10.5)	46.3	(44.3–48.4)	1	(0.9–1.2)	46	(40–54)
50–64yrs	7.9	(7.1–8.8)	74.7	(66.1–84.1)	2.3	(1.9–2.8)	32	(26–41)
> 65yrs	5.8	(4.9–6.9)	237.2	(171–320.7)	4.1	(3.3–5)	58	(40–83)
All ages	7.3	(7.1–7.6)	52	(50.1–54)	1.2	(1.1–1.3)	43	(39–47)

### Descriptive epidemiology

IPD was more common amongst females (53%, 9 096/17 375, gender unknown for 229 cases). Age ranged from 5 years through to 103 years (median 33 years, IQR 25–43). *S*. *pneumoniae* was cultured from CSF in 35% (6 128/17 604) of patients and from blood in 54% (9 537/17 604). The remaining 11% (1 939/17 604) were from either pleural fluid (1 643), peritoneal fluid (123), joint fluid (110) or pus from a deep-seated abscesses (63).

From 2005 to 2008, antimicrobial non-susceptibility of the isolates increased significantly for penicillin (range: 23–29% non-susceptible, MIC≥0.06 μg/ml (p<0.001)), erythromycin (8–11% non-susceptible, MIC≥0.25 μg/ml (p<0.001)) and cotrimoxazole (41–47% non-susceptible, MIC>0.5 μg/ml (p<0.001)) but remained constant for third generation cephalosporins (0.5% non-susceptible, MIC≥1.0 mg/ml (p = 0.028)).

Of 12 547 isolates serotyped (16 were non-typable), the commonest 10 serotypes included serotypes 1, 19A, 4, 14, 23F, 6A, 6B, 3, 8 and 19F (nine of which are included in PCV13). Thirteen patients had a mixed serotype infection. Serotype diversity increased by increasing age with 78% (1 732/2 223) of cases in the 5–19 year; 66% (5 563/8 443) in the 20–49 year; 65% (868/1 328) in the 50–64 year and 61% (337/553) in the ≥65 year age categories included in the commonest 10 serotypes.

**Fig 4:**
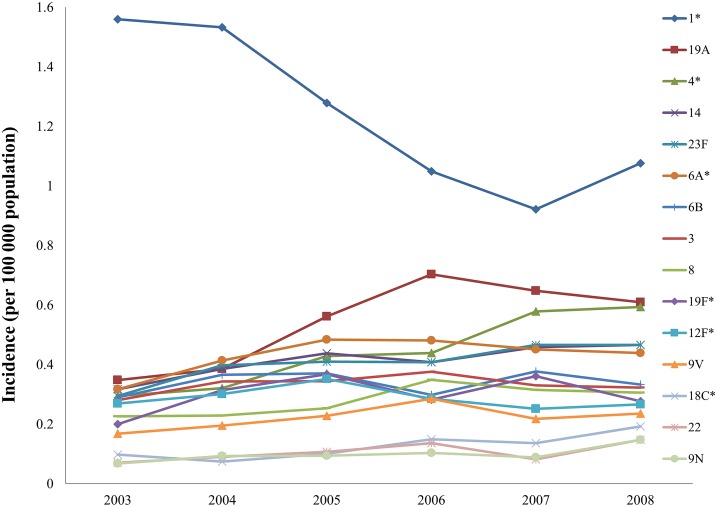
Incidence of invasive pneumococcal disease among persons 5 years and older by the commonest 15 serotypes causing disease, South Africa, 2003–2008 (n = 17 604). Footnote: * Significant changes during the established surveillance period (2005–2008) were seen in serotype 1 (p<0.001) (down), 4 (0.02) (up), 6A (p = 0.0486) (down), 19F (p = 0.0306) (down), 12F (p = 0.0037) (down), 18C (p = 0.0368) (up). Of 17 604 cases of IPD, serotype data were missing and thus imputed for 5 041 cases.

A significant decrease in serotype 1 disease incidence was seen during the surveillance period (p<0.001; 1.14 to 0.7 per 100 000 population), signifying an end to a serotype 1 epidemic that was ongoing prior to the start of the surveillance program. Other significant decreases once the surveillance program was properly established (2005–2008) were seen with serotypes 6A, 19F, 12F, 18A, 15C and 25. Serotypes 4 and 18C were the only serotypes showing significant increases in incidence over time. (Fig 4)

### Comparison of enhanced vs. non-enhanced surveillance sites

Significant differences between cases from enhanced (ES) versus non-enhanced surveillance sites (NESS) included specimen type (25% CSF at ES vs. 44% CSF at NESS, p<0.001, Odds Ratio (OR) 0.4 (95% CI 0.3–0.4)), cotrimoxazole non-susceptibility of the isolates (47% at ES vs. 44% at NESS, p<0.001, OR 1.1 (CI 1.1–1.2)) and percent of vaccine-preventable serotypes (77% serotypes at ES covered by PPV23 vs. 79% at NESS, p = 0.04, OR 0.9 (CI 0.8–1)). The latter two remained significant even after controlling for specimen type. (Table 2)

**Table 2:** Comparison of individuals with invasive pneumococcal disease identified at enhanced versus non-enhanced surveillance sites, South Africa, 2003–2008 (n = 17 604).

	Enhanced sites		Non-enhanced sites		Univariate analysis		Multivariable analysis*	
	n	%	n	%	Odds ratio (95% Confidence interval)	P-value	Odds ratio (95% Confidence interval)	P-value
**Total cases**	8468	48	9136	52				
**Age category**								
5–19 years	1343	16	1609	18	Reference	0.012	Reference	0.152
20–49 years	5884	70	6171	68	1.1 (1.1–1.2)		1.1 (1–1.2)	
50–64 years	886	11	983	11	1.1 (1–1.2)		1.1 (0.9–1.2)	
≥65 years	355	4	373	4	1.1 (1–1.3)		1.0 (0.9–1.3)	
**Specimen type**								
Cerebrospinal Fluid	2106	25	4022	44	Reference	<0.001		
Blood	5683	67	3854	42	2.8 (2.6–3)			
Other	679	8	1260	14	1 (0.9–1.1)			
**Antimicrobial susceptibility**	6 315		6 248					
**Penicillin**								
Non-susceptible	1582	25	1 514	24	1.0 (1–1.1)	0.286		
**Erythromycin**								
Non-susceptible	639	10	641	10	1 (0.9–1.1)	0.795		
**Cotrimoxazole**								
Non-susceptible	2 983	47	2752	44	1.1 (1.1–1.2)	<0.001	1.1 (1.1–1.2)	<0.001
**3**^**rd**^ **Generation Cephalosporin**								
Non-susceptible	34	1	30	1	1.1 (0.7–1.8)	0.647		
**Serotypes (n = 12 563)**	6 315		6 248					
PCV13**								
Vaccine serotypes	4 450	70	4 469	72	0.9 (0.9–1)	0.191		
PPV23***								
Vaccine serotypes	4 874	77	4 916	79	0.9 (0.8–1)	0.043	0.9 (0.8–1)	0.035

Footnote:

*Multivariable analysis was done including all variables with P-values <0.05 and controlling for laboratory syndrome (which accounts for specimen type tested).

**PCV13 (Pneumococcal Conjugate Vaccine-13 valent) serotypes include: 1, 3, 4, 5, 6A, 6B, 7F, 9V, 14, 18C, 19A, 19F, 23F.

***PPV23 (Pneumococcal Polysaccharide Vaccine-23 valent) serotypes include: 1, 2, 3, 4, 5, 6B, 7F, 8, 9N, 9V, 10A, 11A, 12F, 14, 15B, 17F, 18C, 19A, 19F, 20, 22F, 23F, 33F,(6A cross protection is assumed).

### Comparison between HIV-infected vs. HIV-uninfected individuals at ES sites

Eighty-nine percent (4 190/4 734) of people with IPD from ESS with HIV data available were HIV-infected and 37% (1 568/4 190) of these HIV-infected patients were newly diagnosed during this episode of IPD.

On multivariable analysis, age between 20 and 49 years, female sex, bacteremic pneumonia and a cotrimoxazole non-susceptible isolate were significantly more common among HIV-infected than HIV-uninfected IPD patients. HIV-uninfected patients had better survival to discharge, and more underlying conditions (apart from HIV infection) predisposing them to IPD than HIV-infected patients. There was no difference between HIV-infected and–uninfected adults regarding proportion of vaccine-preventable serotypes causing IPD (71% serotypes amongst HIV-infected persons covered by PCV13 vs. 73% amongst HIV-uninfected persons, p = 0.4, OR 0.9 (CI 0.7–1.1) and 84% serotypes amongst HIV-infected persons covered by PPV23 vs. 86% amongst HIV-uninfected persons, p = 0.3, OR 0.9 (CI 0.6–1.1)). (Table 3)

**Table 3:** Comparison of HIV-infected and HIV-uninfected patients (5-years and older) with invasive pneumococcal disease, South Africa, 2003–2008 (n = 4 734).

	HIV-infected		HIV-uninfected		Univariate analysis		Multivariable analysis	
	n	%	n	%	Odds Ratio (95% confidence interval)	P-value	Odds Ratio (95% confidence interval)	P-value
**Total cases**	4 190	89	544	12				
**Age category (n = 4 734)**								
5–19 years	567	14	140	26	Reference	<0.001	Reference	<0.001
20–49 years	3 271	78	274	50	3 (2.4–3.7)		2.9 (2.2–3.9)	
50–64 years	313	8	85	16	0.9 (0.7–1.2)		0.8 (0.6–1.2)	
≥65 years	39	1	45	8	0.2 (0.1–0.3)		0.3 (0.1–0.5)	
**Sex (n = 4 729)**								
Female	2 337	56	224	41	1.8 (1.5–2.2)	<0.001	1.5 (1.2–1.9)	<0.001
**Specimen type (n = 4 734)**								
Cerebrospinal Fluid	875	21	112	21	Reference	0.013		
Blood	3 091	74	386	71	1 (0.8–1.3)			
Other	224	5	46	9	0.6 (0.4–0.9)			
**Clinical syndrome (n = 4 711)**								
Meningitis	1 112	27	148	27	Reference	<0.001	Reference	<0.001
Bacteremic pneumonia	2 653	64	294	54	1.2 (1–1.5)		1.3 (1–1.7)	
Bacteremia without focus	170	4	47	9	0.5 (0.3–0.7)		0.5 (0.3–0.8)	
Other	235	6	52	10	0.6 (0.4–0.9)		0.6 (0.4–0.9)	
**Severity of illness (Pitt bacteremia score) (n = 4 155)**								
0	1 490	41	214	45	Reference		Reference	0.1
1–3	1 815	49	200	42	1.3 (1.1–1.6)	0.009	1.2 (0.9–1.6)	
4–12	375	10	61	13	0.9 (0.7–1.2)		0.8 (0.5–1.2)	
**Predisposing condition (n = 4 734)**								
Yes	790	19	210	39	0.4 (0.3–0.4)	<0.001	0.4 (0.3–0.5)	<0.001
***Predisposing condition specified***								
- ACIP condition#	770	97	206	98	Reference	0.599		
- Other risk factor	20	3	4	2	1.3 (0.5–4)			
**Recurrences of IPD (n = 4 734)**								
Yes	224	5	36	7	0.1 (0.6–1.2)	0.221		
**Outcome (n = 4 710)**								
Died in hospital	1 138	27	107	20	1.9 (1.4–2.5)	<0.001	2 (1.4–2.7)	<0.001
**Antimicrobial susceptibility (n = 3 639)**	3 205		434					
**Penicillin**^								
Non-susceptible	926	29	84	19	1.7 (1.3–2.2)	<0.001		
**Erythromycin**								
Non-susceptible	359	11	37	9	1.4 (1–1.9)	0.093		
**Cotrimoxazole**								
Non-susceptible	1 653	52	157	36	1.9 (1.5–2.3)	<0.001	1.9 (1.5–2.4)	<0.001
**3**^**rd**^ **Generation Cephalosporin**								
Non-susceptible	20	1	2	1	1.4 (0.3–5.8)	0.681		
**Serotypes (n = 3 639)**	3205		434					
PCV13*								
Vaccine types	2272	71	317	73	0.9 (0.7–1.1)	0.353		
PPV23**								
Vaccine types	2685	84	372	86	0.9 (0.6–1.1)	0.301		

Footnote:

^#^ACIP (Advisory Committee on Immunization Practices) condition includes: asplenia; sickle cell anaemia; chronic lung, renal, liver or cardiac disease; diabetes; solid organ transplant; primary immunodeficiency syndromes; immunomodulation therapy; malignancies; head injuries with CSF leak; alcohol dependency and smoking.

^Penicillin non-susceptibility was associated with cotrimoxazole non-susceptibility thus it was not included in the final multivariate model.

*PCV13 (Pneumococcal Conjugate Vaccine-13 valent) serotypes include: 1, 3, 4, 5, 6A, 6B, 7F, 9V, 14, 18C, 19A, 19F, 23F.

**PPV23 (Pneumococcal Polysaccharide Vaccine-23 valent) serotypes include: 1, 2, 3, 4, 5, 6B, 7F, 8, 9N, 9V, 10A, 11A, 12F, 14, 15B, 17F, 18C, 19A, 19F, 20, 22F, 23F, 33F,(6A cross protection is assumed).

## Discussion

In South Africa during the pre-vaccine era, 70% of IPD in adults and older children was caused by serotypes in PCV13 and 85% by PPV23 serotypes. IPD incidence in HIV-infected individuals was 43 times higher than in HIV-uninfected persons (52 per 100 000 vs.1 per 100 000), with HIV-infected adults ≥65 years at highest risk (IPD incidence of 237 per 100 000, 95% CI 171–321). IPD and HIV-coinfection was associated with more bacteremia (with or without pneumonia) and higher in-hospital case fatality than HIV-uninfected cases.

HIV-infection is a major risk factor for IPD, with 90% of IPD amongst South African older children and adults occurring in the 18% of South Africans who are HIV infected.[7, 24] By 2008, HAART coverage amongst HIV-infected South African adults eligible for treatment was 40% [26], this has increased to 79% in recent years.[27] Yet in South Africa, one study showed only a 7% decrease in adult IPD attributable to the increased HAART coverage during this same period.[18] Although HAART reduces the risk of IPD in HIV-infected persons, there seems to be an incomplete restoration of the immune system, necessitating additional measures for preventing IPD in this high risk group. [28–36]

Sex, age and underlying medical conditions are established risk factors for IPD and its more severe outcomes. [37–39] Globally IPD occurs more frequently in males than females however we found that in persons with IPD HIV-infection was associated with female sex. In South Africa, HIV prevalence in females is higher (11.9% in females versus 9.2% in males for 2008) which may in part account for the disparity.[7, 40]

From age 65 years, IPD incidence in both HIV-infected and uninfected persons increases exponentially, however in this age category the incidence amongst HIV-infected persons is 58 times higher than in HIV-uninfected individuals (237 vs. 4 cases per 100 000 population respectively). With the rollout of ARVs, this high-risk category of elderly HIV-infected persons is likely to grow. Similarly, there is a peak of disease in HIV-infected teenagers, which is in stark contrast to their HIV-uninfected peers (81 vs. 0.6 cases per 100 000 population respectively, relative risk of 127). Many of these HIV-infected teens are potentially long term survivors of vertical HIV-transmission, with potential added risk factor of chronic lung disease, whilst a smaller proportion may be recently infected.[41] The HIV-infected teens and elderly are at an even higher risk with the potential development of chronic diseases which further predispose to IPD.

The highest burden of IPD in South African persons five years and older is in the young to middle-aged adult population. The majority (88%) of disease occurred amongst HIV-infected persons; however, 43% of HIV-uninfected persons had other underlying conditions predisposing them to IPD. In our study, as seen in other studies, vaccine serotypes occur at high rates in older children and adults, with 70% of disease caused by PCV13 serotypes and 85% by PPV23 serotypes.[3, 6, 42, 43] This was similar for both HIV-infected and uninfected individuals, despite the individual serotype diversity seen within each group. Although South Africa follows recommendations to vaccinate high risk individuals with PPV23 (including HIV-infected individuals), vaccine uptake is extremely low and no patient in our study had received PPV23.[44, 45]

In South Africa, we recently demonstrated an indirect effect on all serotype IPD incidence, following PCV7 vaccination of infants, of 34% in the 25–44 year age category.[18] However, this indirect effect was less evident amongst those in their early teens and in the elderly (>65 years) (6% reduction in 10–14 years and 14% reduction in 65+ age group)—two groups with high incidences of IPD amongst those HIV-infected. Since 2011, PCV13 has been used in the EPI, and it is expected that the indirect effects of PCV13 on additional serotypes will further decrease the incidence of IPD in older children and adults. However, in HIV-infected populations additional preventative interventions could be considered such as direct vaccination of selected groups with pneumococcal vaccine, increased uptake of the seasonal influenza vaccine and increased use of ART.[46–49]

Previous studies in South Africa reported that HIV-coinfected persons were more likely to be infected with penicillin non-susceptible pneumococci, however when including cotrimoxazole in our multivariate model this finding fell away. Cotrimoxazole non-susceptibility remained a significant finding in HIV-coinfected patients on multivariable analysis; this may be due to the high use of cotrimoxazole prophylaxis in preventing opportunistic infections in HIV-infected patients in South Africa.[50]

One important limitation of surveillance studies is that IPD incidence may be underestimated.[51] Our case definition relies on the specimen taking practices of clinicians and the health seeking behavior of the patients, which have both been found to vary greatly across our study population.[52, 53] In many of the rural areas reporting of blood culture results are often delayed, therefore if patients do seek medical care, clinicians often treat those with a suspected bacteremia empirically without laboratory investigations to confirm their diagnosis. These cases would not have been reflected in this study and are therefore excluded from our incidence calculations. A study during the same time period confined to a single site in South Africa with a relatively well-defined population and adequate blood culturing practices, reported IPD incidence in adults of 58 per 100 000 population.[28] Therefore the true incidence of IPD in South African adults and older children likely lies above the 7 per 100 000 reflected here. An assumption was made for 29% (5 041/17 604) of IPD isolates that distribution of serotypes by age-category and province was similar amongst isolates that were available and those that were missing when calculating incidence by serotype. In addition, these missing isolates could influence the distribution of antimicrobial susceptibility data. HIV testing was not done routinely for surveillance purposes and patient HIV serostatus was only available at ES sites—and only for 68% of the patients with medical records available. Only a few parameters were available for comparing cases from ES versus non-enhanced sites, and although the main differences were found in specimen type and cotrimoxazole non-susceptibility there may be more unmeasured differences which we were unable to account for which could have influenced our model comparing HIV-infected to HIV-uninfected patients with IPD. ASSA2003 denominators for HIV-infected individuals tended to underestimate the number of HIV-infected individuals in the teenage and elderly populations, therefore we used a modified version of the ASSA2003 model, Thembisa 1.7v3, which accounts for the survival benefits of the rollout of HAART especially amongst the teenagers and elderly.[25, 54] This gave more plausible results for IPD incidence amongst the teenager and elderly HIV-infected population but may still be an underestimate of true IPD incidence.

In summary, 70–85% of adult IPD in the pre-PCV era were vaccine serotypes and 93% of cases had recognized risk factors (including HIV-infection) for pneumococcal vaccination. These data describe the epidemiology of IPD amongst HIV-infected and -uninfected adults during the pre-PCV era and provide a robust baseline to calculate the indirect effect of PCV in future studies.

## References

[1] Janoff EN, Breiman RF, Daley CL, Hopewell PC. Pneumococcal disease during HIV infection. Epidemiologic, clinical, and immunologic perspectives. Ann Intern Med. 1992;117(4):314–24. 1637028 10.7326/0003-4819-117-4-314

[2] Gilks CF, Ojoo SA, Ojoo JC, Brindle RJ, Paul J, Batchelor BI, et al Invasive pneumococcal disease in a cohort of predominantly HIV-1 infected female sex-workers in Nairobi, Kenya. Lancet. 1996;347(9003):718–23. 8602001 10.1016/s0140-6736(96)90076-8

[3] Jones N, Huebner R, Khoosal M, Crewe-Brown H, Klugman K. The impact of HIV on Streptococcus pneumoniae bacteraemia in a South African population. AIDS. 1998;12(16):2177–84. 9833859 10.1097/00002030-199816000-00013

[4] Feikin DR, Jagero G, Aura B, Bigogo GM, Oundo J, Beall BW, et al High rate of pneumococcal bacteremia in a prospective cohort of older children and adults in an area of high HIV prevalence in rural western Kenya. BMC Infect Dis. 2010;10:186 10.1186/1471-2334-10-186 20573224 PMC2901359

[5] French N, Nakiyingi J, Carpenter LM, Lugada E, Watera C, Moi K, et al 23-valent pneumococcal polysaccharide vaccine in HIV-1-infected Ugandan adults: double-blind, randomised and placebo controlled trial. Lancet. 2000;355(9221):2106–11. 10902624 10.1016/s0140-6736(00)02377-1

[6] Nuorti JP, Butler JC, Gelling L, Kool JL, Reingold AL, Vugia DJ. Epidemiologic relation between HIV and invasive pneumococcal disease in San Francisco County, California. Ann Intern Med. 2000;132(3):182–90. 10651598 10.7326/0003-4819-132-3-200002010-00003

[7] ASSA2003 AIDS and demographic model [Internet]. 2005. Available from: http://aids.actuarialsociety.org.za/ASSA2003-model-3165.htm.

[8] Yin Z, Rice BD, Waight P, Miller E, George R, Brown AE, et al Invasive pneumococcal disease among HIV-positive individuals, 2000–2009. AIDS. 2012;26(1):87–94. 10.1097/QAD.0b013e32834dcf27 . 22008657

[9] Hibbs JR, Douglas JM Jr., Judson FN, McGill WL, Rietmeijer CA, Janoff EN. Prevalence of human immunodeficiency virus infection, mortality rate, and serogroup distribution among patients with pneumococcal bacteremia at Denver General Hospital, 1984–1994. Clin Infect Dis. 1997;25(2):195–9. 9332509 10.1086/514538

[10] Karstaedt AS, Khoosal M, Crewe-Brown HH. Pneumococcal bacteremia in adults in Soweto, South Africa, during the course of a decade. Clin Infect Dis. 2001;33(5):610–4. 11477524 10.1086/322589

[11] Crewe-Brown HH, Karstaedt AS, Saunders GL, Khoosal M, Jones N, Wasas A, et al Streptococcus pneumoniae blood culture isolates from patients with and without human immunodeficiency virus infection: alterations in penicillin susceptibilities and in serogroups or serotypes. Clin Infect Dis. 1997;25(5):1165–72. 9402377 10.1086/516104

[12] Crowther-Gibson P, Cohen C, Klugman KP, de GL, von GA. Risk factors for multidrug-resistant invasive pneumococcal disease in South Africa, a setting with high HIV prevalence, in the prevaccine era from 2003 to 2008. Antimicrob Agents Chemother. 2012;56(10):5088–95. 22802256 10.1128/AAC.06463-11 PMC3457358

[13] Von Mollendorf C, Cohen C, Tempia S, Meiring S, De Gouveia L, Quan V, et al Epidemiology of serotype 1 invasive pneumococcal disease in all ages in South Africa, 2003–2013 Emerg Infect Dis. 2016;22(2). 10.3201/eid2202.150967 PMC4734528 26812214

[14] Burgos J, Penaranda M, Payeras A, Villoslada A, Curran A, Garau M, et al Invasive pneumococcal disease in HIV-infected adults: clinical changes after the introduction of the pneumococcal conjugate vaccine in children. J Acquir Immune Defic Syndr. 2012;59(1):31–8. 10.1097/QAI.0b013e31823d0f5f 22156821

[15] French N, Gordon SB, Mwalukomo T, White SA, Mwafulirwa G, Longwe H, et al A trial of a 7-valent pneumococcal conjugate vaccine in HIV-infected adults. N Engl J Med. 2010;362(9):812–22. 10.1056/NEJMoa0903029 20200385 PMC2873559

[16] Whitney CG, Farley MM, Hadler J, Harrison LH, Bennett NM, Lynfield R, et al Decline in invasive pneumococcal disease after the introduction of protein-polysaccharide conjugate vaccine. N Engl J Med. 2003;348(18):1737–46. 12724479 10.1056/NEJMoa022823

[17] Pilishvili T, Lexau C, Farley MM, Hadler J, Harrison LH, Bennett NM, et al Sustained reductions in invasive pneumococcal disease in the era of conjugate vaccine. J Infect Dis. 2010;201(1):32–41. 10.1086/648593 19947881

[18] von Gottberg A, de Gouveia L, Tempia S, Quan V, Meiring S, von Mollendorf C, et al Effects of vaccination on invasive pneumococcal disease in South Africa. N Engl J Med. 2014;371(20):1889–99. 10.1056/NEJMoa1401914 . 25386897

[19] Cohen AL, Harrison LH, Farley MM, Reingold AL, Hadler J, Schaffner W, et al Prevention of invasive pneumococcal disease among HIV-infected adults in the era of childhood pneumococcal immunization. AIDS. 2010;24(14):2253–62. 10.1097/QAD.0b013e32833d46fd 20671543

[20] GERMS-SA Annual Report 2008 [Internet]. 2009. Available from: http://nicd.ac.za/?page=germs-sa&id=97.

[21] Clinical and Laboratory Standards Institute. Performance standards for antimicrobial susceptibility testing In: Wayne PA, editor. Eighteenth informational supplement: CLSI; 2008.

[22] Paterson DL, Hujer KM, Hujer AM, Yeiser B, Bonomo MD, Rice LB, et al Extended-spectrum beta-lactamases in Klebsiella pneumoniae bloodstream isolates from seven countries: dominance and widespread prevalence of SHV- and CTX-M-type beta-lactamases. Antimicrob Agent Chemother. 2003;47(11):3554–60. 14576117 10.1128/AAC.47.11.3554-3560.2003 PMC253771

[23] Centers for Disease Control and Prevention (CDC). Use of PCV13 and PPV23 vaccines for adults with immunocompromising conditions. MMWR RecommRep. 2012;61(40):816–9. 23051612

[24] Mid-year population estimates, South Africa, 2008 [Internet]. 2008. Available from: http://www.statssa.gov.za/publications/P0302/P03033010.pdf.

[25] Johnson LF, Rehle TM, Jooste S, Bekker LG. Rates of HIV testing and diagnosis in South Africa: successes and challenges. AIDS. 2015;29(11):1401–9. 10.1097/QAD.0000000000000721 . 26091299

[26] Adam MA, Johnson LF. Estimation of adult antiretroviral treatment coverage in South Africa. S Afr Med J. 2009;99(9):661–7. . 20073293

[27] Johnson LF. Access to antiretroviral treatment in South Africa, 2004–2011. South Afr J HIV Med. 2012;13(1):22–7. 10.4102/sajhivmed.v18i1.694 PMC5843157 29568630

[28] Nunes MC, von Gottberg A, de Gouveia L, Cohen C, Kuwanda L, Karstaedt AS, et al Persistent high burden of invasive pneumococcal disease in South African HIV-infected adults in the era of an antiretroviral treatment program. PloS one. 2011;6(11):e27929 10.1371/journal.pone.0027929 22140487 PMC3225377

[29] Palella FJ Jr., Delaney KM, Moorman AC, Loveless MO, Fuhrer J, Satten GA, et al Declining morbidity and mortality among patients with advanced human immunodeficiency virus infection. HIV Outpatient Study Investigators. N Engl J Med. 1998;338(13):853–60. 10.1056/NEJM199803263381301 . 9516219

[30] Everett DB, Mukaka M, Denis B, Gordon SB, Carrol ED, van Oosterhout JJ, et al Ten years of surveillance for invasive Streptococcus pneumoniae during the era of antiretroviral scale-up and cotrimoxazole prophylaxis in Malawi. PloS one. 2011;6(3):e17765 10.1371/journal.pone.0017765 21423577 PMC3058053

[31] Mayanja BN, Todd J, Hughes P, Van der Paal L, Mugisha JO, Atuhumuza E, et al Septicaemia in a population-based HIV clinical cohort in rural Uganda, 1996–2007: incidence, aetiology, antimicrobial drug resistance and impact of antiretroviral therapy. Trop Med Int Health. 2010;15(6):697–705. 10.1111/j.1365-3156.2010.02528.x . 20406428

[32] Heffernan RT, Barrett NL, Gallagher KM, Hadler JL, Harrison LH, Reingold AL, et al Declining incidence of invasive Streptococcus pneumoniae infections among persons with AIDS in an era of highly active antiretroviral therapy, 1995–2000. J Infect Dis. 2005;191(12):2038–45. 10.1086/430356 . 15897989

[33] Glennie SJ, Sepako E, Mzinza D, Harawa V, Miles DJ, Jambo KC, et al Impaired CD4 T cell memory response to Streptococcus pneumoniae precedes CD4 T cell depletion in HIV-infected Malawian adults. PloS one. 2011;6(9):e25610 10.1371/journal.pone.0025610 21980502 PMC3181344

[34] Sepako E, Glennie SJ, Jambo KC, Mzinza D, Iwajomo OH, Banda D, et al Incomplete recovery of pneumococcal CD4 T cell immunity after initiation of antiretroviral therapy in HIV-infected malawian adults. PloS one. 2014;9(6):e100640 10.1371/journal.pone.0100640 24959834 PMC4069109

[35] Jordano Q, Falco V, Almirante B, Planes AM, del Valle O, Ribera E, et al Invasive pneumococcal disease in patients infected with HIV: still a threat in the era of highly active antiretroviral therapy. Clin Infect Dis. 2004;38(11):1623–8. 10.1086/420933 . 15156452

[36] Siemieniuk RA, Gregson DB, Gill MJ. The persisting burden of invasive pneumococcal disease in HIV patients: an observational cohort study. BMC Infect Dis. 2011;11:314 10.1186/1471-2334-11-314 22078162 PMC3226630

[37] Feikin DR, Schuchat A, Kolczak M, Barrett NL, Harrison LH, Lefkowitz L, et al Mortality from invasive pneumococcal pneumonia in the era of antibiotic resistance, 1995–1997. Am J Public Health. 2000;90(2):223–9. 10667183 10.2105/ajph.90.2.223 PMC1446155

[38] Moroney JF, Fiore AE, Harrison LH, Patterson JE, Farley MM, Jorgensen JH, et al Clinical outcomes of bacteremic pneumococcal pneumonia in the era of antibiotic resistance. Clin Infect Dis. 2001;33(6):797–805. 10.1086/322623 . 11512085

[39] Yu VL, Chiou CC, Feldman C, Ortqvist A, Rello J, Morris AJ, et al An international prospective study of pneumococcal bacteremia: correlation with in vitro resistance, antibiotics administered, and clinical outcome. Clin Infect Dis. 2003;37(2):230–7. 10.1086/377534 . 12856216

[40] Buie KA, Klugman KP, von G, A., Perovic O, Karstaedt A, Crewe-Brown HH, et al Gender as a risk factor for both antibiotic resistance and infection with pediatric serogroups/serotypes, in HIV-infected and -uninfected adults with pneumococcal bacteremia. J Infect Dis. 2004;189(11):1996–2000. 15143465 10.1086/386548

[41] Ferrand RA, Desai SR, Hopkins C, Elston CM, Copley SJ, Nathoo K, et al Chronic lung disease in adolescents with delayed diagnosis of vertically acquired HIV infection. Clin Infect Dis. 2012;55(1):145–52. 10.1093/cid/cis271 22474177 PMC3369563

[42] Dworkin MS, Ward JW, Hanson DL, Jones JL, Kaplan JE. Pneumococcal disease among human immunodeficiency virus-infected persons: incidence, risk factors, and impact of vaccination. Clin Infect Dis. 2001;32(5):794–800. 11229848 10.1086/319218

[43] Feikin DR, Klugman KP, Facklam RR, Zell ER, Schuchat A, Whitney CG. Increased prevalence of pediatric pneumococcal serotypes in elderly adults. Clin Infect Dis. 2005;41(4):481–7. 16028155 10.1086/432015

[44] SAMA-SA Pulmonology Society Working Group. Adult pneumococcal vaccination guideline S Afr Med J. 1999;89(11 Suppl):1222–30. . 10599303

[45] Souter J. An update on pneumococcal vaccination in children and adults. S Afr Pharma J. 2014;81(2):15–8.

[46] Burgos J, Larrosa MN, Martinez A, Belmonte J, Gonzalez-Lopez J, Rello J, et al Impact of influenza season and environmental factors on the clinical presentation and outcome of invasive pneumococcal disease. Eur J Clin Microbiol Infect Dis. 2015;34(1):177–86. 10.1007/s10096-014-2221-9 . 25109886

[47] Bhorat AE, Madhi SA, Laudat F, Sundaraiyer V, Gurtman A, Jansen KU, et al Immunogenicity and safety of the 13-valent pneumococcal conjugate vaccine in HIV-infected individuals naive to pneumococcal vaccination. AIDS. 2015;29(11):1345–54. 10.1097/QAD.0000000000000689 25888646 PMC4521829

[48] Ranieri R, Veronelli A, Santambrogio C, Pontiroli AE. Impact of influenza vaccine on response to vaccination with pneumococcal vaccine in HIV patients. AIDS Res Hum Retroviruses. 2005;21(5):407–9. 10.1089/aid.2005.21.407 . 15929703

[49] Atashili J, Kalilani L, Adimora AA. Efficacy and clinical effectiveness of influenza vaccines in HIV-infected individuals: a meta-analysis. BMC Infect Dis. 2006;6:138 10.1186/1471-2334-6-138 16965629 PMC1574329

[50] Soeters HM, von Gottberg A, Cohen C, Quan V, Klugman KP. Trimethoprim-sulfamethoxazole prophylaxis and antibiotic nonsusceptibility in invasive pneumococcal disease. Antimicrob Agent Chemother. 2012;56(3):1602–5. 10.1128/AAC.05813-11 22232291 PMC3294938

[51] Bar-Zeev N, Mtunthama N, Gordon SB, Mwafulirwa G, French N. Minimum incidence of adult invasive pneumococcal disease in Blantyre, Malawi an urban African setting: a hospital based prospective cohort study. PloS one. 2015;10(6):e0128738 10.1371/journal.pone.0128738 26039077 PMC4454543

[52] Cheyip M, Cohen C, von Gottberg A, Govender N, Keddy K, GERMS-SA. Collection rates for blood culture and cerebrospinal fluid specimens and estimated burden of disease due to invasive respiratory, meningeal and enteric bacterial pathogens in South Africa. University of the Witwatersrand School of Public Health Annual Research Day; Johannesburg, South Africa2007.

[53] Wong KK, von Mollendorf C, Martinson NA, Norris SA, Tempia S, Walaza S, et al Healthcare utilization for common infectious disease syndromes in Soweto and Klerksdorp, South Africa. BMC Infect Dis. 2015;In press. 10.11604/pamj.2018.30.271.14477 PMC6317391 30637056

[54] Initial observations on the comparison of the 2005 HSRC household HIV prevalence and behaviour survey amongst estimates from the ASSA2003 AIDS and Demographic model [Internet]. 2006. Available from: www.actuarialsociety.co.za/applications/cms/documents/file_build.asp?id=100000119.

